# Dietary pattern and nutritional status of female adolescents in Amai Secondary School, Delta State, Nigeria

**DOI:** 10.11604/pamj.2021.38.32.15824

**Published:** 2021-01-13

**Authors:** Otovwe Agofure, Stella Odjimogho, Oghenenioborue Okandeji-Barry, Votapwa Moses

**Affiliations:** 1 Department of Public and Community Health, Novena University Ogume, Delta State, Nigeria,; 2 Department of Opthometry, University of Benin, Edo State, Nigeria,; 3 Department of Health and Social Care Management, London School of Management Education, London, United Kingdom

**Keywords:** Female, adolescent, nutritional status, snacks, taste, Nigeria, students, body weight, diet, carbohydrates

## Abstract

**Introduction:**

there is an existing variability in eating patterns between adolescents and children. Consequently, with the adoption of westernised way of life, this translates to a change in eating habits and food choices. This study was designed to investigate the dietary patterns and nutritional status of the female adolescents in Amai Secondary Commercial School, Delta State, Nigeria.

**Methods:**

a cross-sectional study was conducted among 201 adolescent female students (12-18 years) using simple random sampling techniques. A self-administered questionnaire was used to obtain the information while anthropometric measurement was carried out to obtain the height and weight of the respondents. Data generated were analysed using SPSS version 22.0.

**Results:**

majority of the respondents 129 (64.20%) were between the ages of 16-18 years. Measurement of nutritional status confirmed that 46.80% and 31.80% of the respondents were underweight and healthy weight respectively. In addition, the dietary pattern of the respondents demonstrated that they consumed proteinous, carbohydrate, and snacks occasionally. Factors that motivated respondents for good food choices include; nutritional status 71 (35.30%), taste 54 (26.90%), and popularity 15 (7.50%).

**Conclusion:**

under nutrition remains a challenge among rural adolescent girls in Amai community. Therefore sustained strategic nutritional campaigns should be carried out among female adolescents in Amai community in order to improve their nutritional status.

## Introduction

The World Health Organization defines an adolescent as a person between ages 10 and 19 years who is undergoing a transitional and developmental phase between childhood and adulthood [1]. Adolescence is one of the fastest growth periods of a person's life. As a result of the physical, psychological, hormonal, cognitive and social transformation changes that occur during this growth period, the body’s nutritional needs, eating habits and food choices fluctuate. These changes have implications towards the alarming rate of non-communicable disease including obesity, hypercholesteramia, high glucose levels which are emerging public health problems globally especially in developing countries [2, 3]. An adolescent eating pattern varies from that of children. The increased academic and socio-economic demands associated with this growth stage results in skipping meals, increased consumption of refined and “fast” foods and decrease in consumption of fruits and vegetables [4-6]. This was corroborated in a study which revealed that adolescents do not conform to a regular dietary pattern of 3 meals per day, as over 50% eat at least 5 times daily [3, 7].

Further, increased attention to body image and appearance by adolescent girls impinges negatively on their nutritional pattern. Dissatisfaction with body image which refers to the feelings and reflections of an adolescent girl towards her body translates to deteriorating nutritional consequences. These dietary patterns predispose adolescents to cardiovascular risk factors which sets the stage for early onset of non-communicable diseases which is already assuming an alarming rate among young people who are key drivers of the socio-economic activities of developing countries [3, 8]. The adolescence phase is a critical growth stage which offers a strategic window of opportunity to shape and consolidate healthy eating and lifestyle behaviours among young people. For increased physical growth, psychosocial development, optimal cognitive performance and prevention of cardiovascular diseases, it is pertinent to investigate in-depth the dietary pattern and nutritional status of adolescent girls in a rural community. This study was therefore designed for determining the dietary pattern and nutritional status of the female adolescent in Amai Secondary Commercial School Delta State, Nigeria.

## Methods

**Study design and setting:** the study was a descriptive cross-sectional survey that investigated the dietary pattern and nutritional status of the female adolescent in Amai Secondary Commercial School, Delta state. The study was conducted in Amai Mixed Secondary Commercial School in Amai community in Ukwuani Local Government Area of Delta State Nigeria. The school is a mixed school comprising both males and females and the school was established to serve the five clans of Amai Kingdom.

**Sample size and sampling technique:** formula for sample size determination:

$$n=\frac{N}{1+N\sigma^{2}}$$

N = 340 population of girls in JSS1-SS2

= 0.005 expected frequency

$$846/(1+(846\times \left[\left(0.005\right)\right]↑2)$$

$$n=\frac{846}{1+(846\times 0.0025)}$$

$$n=\frac{846}{1+2.115}$$

$$n=\frac{846}{3.115}$$

n =183.7

n = 184

Therefore the sample size was adjusted to 201 to cover for attrition and non-response. A simple random sampling technique was used to administer the questionnaire across the classes from JSS1-SS2 after the proportionate allocation of each of the students from each class to generate the required sample size.

**Method and instrument for data collection:** data were collected between May and June 2016, using a semi-structured self-administered questionnaire that measured the dietary pattern and nutritional status of the female adolescent in Amai secondary commercial school. The questionnaire was divided into 2 parts: the first part sought information on sociodemographic data, such as the age of participants, educational status of father and mother, the occupation of father, number of people in the household. The second part consisted of questions relating to eating patterns and habits which included questions on the number of meals eaten per day and habit of skipping meals. The number of meals as defined here means taking breakfast, lunch and dinner, and any other meal for those who eat more than three times daily. A snack is defined as light food and/or a drink that is consumed outside the main meals of breakfast, lunch and dinner while skipping meals involves respondents who did not take any of the regular meals of breakfast, lunch and dinner very often, often or sometimes. In the context of the study “very often” means taking of the listed meals at least 4 times a week, while “often” means at least 2-3 times in a week and “sometimes” means 1-2 times in every week. Other information collected includes data on food frequency, such as carbohydrates, proteins, fats and oils, fruits and vegetables, drinks and fast foods.

**Statistical methods:** the anthropometric measurement such as height and weight of the respondents were taken. Their heights were taken using a standard stadiometer and a portable calibrated weighing scale to the nearest 0.1kg was used to measure the weight of the respondents. Both measurements were taken using standard procedures, as described by the World Health Organisation [9] and a previous study [10]. Body mass index (BMI) was calculated as the ratio between the subjects’ weight in kilograms and the square of the respondent’s height in meters. The BMI was graded as <18.5kg/m^2^ for underweight, 18.5-24.9 kg/m^2^ as normal or healthy weight, and >25 kg/m^2^ as overweight. Validity was achieved by extensive literature review and subjecting the work to the expertise of professional critics. The instrument was pretested at Obiaruku Grammar School to improve validity. Furthermore, some part of the instrument was adapted from a previous study [10]. Cronbach alpha was used to test the reliability of the instrument which gave a score of 0.792. Data analysis was performed on SPSS for Windows version 22.0 (IBM Corp, Armonk, NY, USA). The analysed data were presented using descriptive statistics.

**Ethical approval:** as a school-based study, institutional ethical permission was provided by administrative head of the Amai Secondary Commercial School to carry out the study.

## Results

**Socio-demographic characteristics of the respondents:** the majority of the respondents 73 (36.30%) were age 12-15 years and most 141 (70.10%) were Christians. In addition, 129 (64.20%) of the respondents lived with their parents while 91 (45.30%) and 60 (29.30%) of the respondents had parents who attained a secondary and tertiary level of education respectively as shown in Table 1.

**Table 1:** socio-demographic characteristics of the respondents (n=201)

Variable	Frequency	Percentage
**Age**		
12-15	73	36.30
16-18	128	63.70
**Religion**		
Christianity	141	70.10
Islam	18	9.0
Traditional	32	15.90
Others	10	5.0
**Living with**		
Both parents	129	64.20
Mother only	35	17.40
Father only	20	10.0
Others	17	8.50
**Educational Level of parents**		
Primary	28	13.90
Secondary	91	45.30
Tertiary	60	29.90
No formal Education	22	10.90

**Knowledge of dietary habits:** the majority of the respondents 174 (86.60%) affirmed to have knowledge of the word balanced diet while 175 (87.10%) knew that taking a balanced diet is good and more than half 105 (52.20%) agreed that taking balanced diet will make them live longer. Furthermore, more than half of the respondents 114 (56.70%) reported eating thrice a day while 41 (20.40%) ate twice daily and 25 (12.40%) ate four times daily as shown in Table 2.

**Table 2:** knowledge of good dietary habits

Variable	Frequency	Percentage
**Awareness of balance diet**		
Yes	174	86.60
No	27	13.40
**It is good to take a balanced diet**		
Yes	175	87.10
No	26	12.90
**Eating a balanced diet is good for the body**		
True	172	85.60
False	29	14.40
**Taking a balanced diet will make you live longer and healthier**		
Strongly agree	105	52.20
Agree	49	24.40
Disagree	11	5.50
Strongly disagree	10	5.0
I don’t Know	26	12.90
**How many times do you eat daily**		
1	10	5.0
2	41	20.40
3	114	56.70
4	25	12.40
5	10	5.0
Others	1	0.50

**Dietary pattern and habits among respondents:** less than half of the respondents 83 (41.30%) affirmed that they eat eggs sometimes while 67 (33.30%) affirmed that they take milk sometimes and 71 (35.30%) affirmed that they eat sweet sometimes. Also, less than half 91 (45.30%) confirm that they take ice cream sometimes while 78 (38.80%) of the respondents agreed that they take fruits sometimes and almost half 96 (47.80%) confirmed that they take garri/eba very often. In addition 69 (34.40%) of the respondents observe three square meals sometimes while 77 (38.30%) established that they take breakfast very often and almost half of the respondents 97 (48.30%) confirm that they eat fruits after meals sometimes Table 3.

**Table 3:** dietary pattern and habits among the respondents

How often do you eat the following	Very Often		Often		Sometimes		Never	
	F	%	F	%	F	%	F	%
Egg	40	19.90	67	33.30	83	41.30	11	5.50
Hot drink	41	20.40	47	23.40	54	26.90	59	29.40
Milk	63	31.30	64	31.80	67	33.30	7	3.50
Meat	112	55.70	50	24.90	38	18.90	1	0.50
Sweet	61	30.30	58	28.90	71	35.30	11	5.50
Ice cream	46	22.90	52	25.90	91	45.30	12	6.0
Snacks	52	25.90	63	31.30	64	31.80	22	10.90
Coffee	30	14.90	32	15.90	57	28.40	82	40.80
Spicy foods	49	24.40	69	34.30	68	33.80	15	7.50
Cold drinks	65	32.30	68	33.80	60	29.90	8	4.0
Fruits	60	29.90	61	30.30	78	38.80	2	1.0
Chips	39	19.40	60	29.90	90	44.80	12	6.0
Eba/Garri	96	47.80	52	25.90	48	23.90	5	2.50
Akpu/Fufu	80	39.80	40	19.90	73	36.30	8	4.0
Pounded Yam/Yam	48	23.90	58	28.90	79	39.30	16	8.0
Bean cake/Akara	62	30.80	59	29.40	70	34.80	10	5.0
Moi-Moi	56	27.90	67	33.30	72	35.80	6	3.0
Potato	41	20.40	61	30.30	92	45.80	7	3.50
Meat pie	55	27.40	65	32.30	73	36.30	8	4.0
Cake	66	32.80	55	27.40	78	38.80	2	1.0
Chocolate	63	31.30	70	34.80	52	25.90	16	8.0
Biscuits	90	44.80	51	25.40	58	28.90	2	1.0
Observe three square meals	62	30.80	61	30.30	69	34.40	9	4.50
Take your breakfast	77	38.30	52	25.90	67	33.30	5	2.50
Take fruits after meal	39	19.40	46	22.90	97	48.30	19	9.50
Skip meals	46	22.90	50	24.90	84	41.80	21	10.40

**Factors that motivate choices of food:** the following factors motivate the nutritional meal choices of respondents including nutritional values 71 (35.30%), taste 54 (26.90%), the popularity of the food 15 (7.50%), and cost 21 (10.40%). Others were time 14 (7.0%), preparation 8 (8.0%) and appearance 14 (7.0%). In the order of importance, breakfast, lunch and dinner is the most important meal of the day to [49 (24.40%)], [44 (21.90%)] and [29 (14.40%)] respectively as shown in Table 4.

**Table 4:** factors that motivate food choices/anthropometric features of respondents

Variable	Frequency	Percentage
**The motivation for good food choices are**		
Taste	54	26.90%
Nutritional values	71	35.30%
Popularity	15	7.50%
Cost	21	10.40%
Time	14	7.0%
Preparation	8	4.0%
Appearance	14	7.0%
Other reasons	4	2.0%
**The most important meal for you in a day is;**		
Breakfast	49	24.40%
Lunch	44	21.90%
Dinner	29	14.40%
All of them	79	39.30%
Underweight	94	46.80
Healthy weight	64	31.80
Overweight	43	21.40

## Discussion

The rapid growth involved in the adolescence period requires and demands increased nutritional requirements for a healthy living and quality health of life among adolescents. A similar report revealed that lifestyle and dietary habit may either have a negative influence on adolescent health status or rather contribute to the quality of health during progression to adulthood [11].

According to the study more of the respondents were between the ages of 16-18 years. This finding is similar to a study conducted in South-South Nigeria where the students were between the ages of 17-19 years [3]; but different from a study conducted in South-West Nigeria where the majority of the students were between the ages of 12-14 years [12]. In addition, more of the respondents were living with both parents and their parents have attained at least secondary education. This is similar to previous findings both in India and Nigeria [3, 10, 11].

Further, almost half of the respondents were underweight followed by normal or healthy weight and overweight. This finding was different from the study in Ibadan Nigeria and in a rural area of West Bengal India where most of the students had a normal or healthy weight, followed by underweight and overweight [11, 13]. The difference in the findings could be attributed to the rural setting of the study population which reveals that under nutrition is probably still prevalent among adolescent girls located in rural areas.

Furthermore, the respondents had knowledge of the word “balanced diet” and its importance towards maintaining a healthy life. However, this knowledge did not translate into their nutritional status, revealing that apart from knowledge, there are other factors influencing intake of balanced diet among adolescents’ girls. In addition, more than half of the respondents ate three times daily. This finding varies from the study in South-West Nigeria where the meal pattern showed that more of the respondents consumed both breakfast and dinner compared to lunch [12]. This observed difference in the findings probably might be a result of after school extra-curricular activities being offered in most secondary schools in Nigeria which makes them skip lunch which, to the best of the researcher’s knowledge, is not offered in Amai Secondary School.

According to the reported dietary pattern the respondents ate proteinous food such as egg, milk, meat, Bean cake/Akara and MoiMoi (local food made with beans). This is similar to previous studies in Nigeria where the respondents ate proteinous food such as milk and dairy products and meat and alternatives [3, 12]. Similarly, the respondents also consumed carbohydrate-laden food such as Eba/Garri, Akpu/Fufu and pounded Yam/Yam. This is also similar to the study in Edo State Nigeria where the respondents ate a lot of starchy food [3]. This finding was not surprising as carbohydrate-laden meal remains a staple meal in Nigeria. In addition, the respondents also ate snacks and sometimes skip meals. This is also similar to previous findings [3]. Some of the factors that motivated the respondents on their choice of the meal included the perceived nutritional value of the meal, taste, popularity of the food, and economic capacity.

The implication of the findings is that if proper nutritional campaign is not carried out in the study area, adolescent girls who are undernourished might grow up to have challenges with their reproduction or in more severe cases might give birth to low birth weight babies. Strengths of the Study: The strength of this study is that the study was conducted in the rural part of Delta State where there is a paucity of data on the nutritional status of adolescent girls. Limitations of the study: The study may be limited as responses on the dietary pattern of the respondents, food choices, and other collected data were based solely on the responses of the respondents which might be subject to recall bias.

## Conclusion

Despite the reported knowledge on the importance of balanced diet among the respondents, almost half of them were underweight as evidenced by their Body Mass Index Classification. More sustainable, holistic and culturally-sensitive interventions and programmes should be targeted towards adolescent girls in the study area in order to improve their nutritional status and quality of life.

### What is known about this topic

- Adolescence is one of the fastest growth periods of a person's life and change in lifestyle may affect eating habits and food choices;
- Poor eating habits are often observed among adolescent girls because most of them are interested in diets that will make them thinner and improve their complexions;
- Globally overweight and obesity have been reported among adolescents.

### What this study adds

- The study documented under nutrition among adolescent girls which could have severe implication in their future reproductive outcomes;
- Knowledge was not enough to influence choice of meals among the adolescent girls;
- Taste, popularity of food, cost, time, preparations and appearance were identified factors that motivated choices of food among adolescent girls.
